# 5-*tert*-Butyl-2-[5-(5-*tert*-butyl-1,3-benzoxazol-2-yl)-2-thien­yl]-1,3-benzoxazole

**DOI:** 10.1107/S1600536808037665

**Published:** 2008-11-22

**Authors:** Yu-Feng Li, Lin-Tong Wang, Fang-Fang Jian

**Affiliations:** a Microscale Science Institute, Department of Chemistry and Chemical Engineering, Weifang University, Weifang 261061, People’s Republic of China; b Microscale Science Institute, Weifang University, Weifang 261061, People’s Republic of China

## Abstract

The title compound, C_26_H_26_N_2_O_2_S, was prepared by the reaction of thio­phene-2,5-dicarboxylic acid and 2-amino-4-*tert*-butyl­phenol. One of the *tert*-butyl groups is disordered over two conformations, with occupancies of 0.539 (1) and 0.461 (2). The two 1,3-benzoxazole rings are almost planar, with dihedral angles of 0.83 (18) and 1.64 (17)° between the five- and six-membered rings. The thio­phene ring makes dihedral angles of 21.54 (19) and 4.49 (18)° with the planes of the five-membered oxazole rings. The crystal packing is controlled by π–π stacking inter­actions involving the thio­phene and benzene rings, with a centroid–centroid distance of 3.748 (2) Å.

## Related literature

For background on fluorescent whitening agents, see: Chen *et al.* (2008 ▶). For a related structure, see: Cowley *et al.* (2002 ▶). 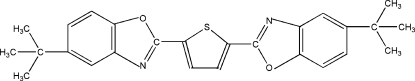


## Experimental

### 

#### Crystal data

C_26_H_26_N_2_O_2_S
*M* *_r_* = 430.56
Triclinic, 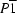

*a* = 6.0852 (12) Å
*b* = 11.520 (2) Å
*c* = 16.986 (3) Å
α = 72.79 (3)°
β = 88.88 (3)°
γ = 79.32 (3)°
*V* = 1116.9 (4) Å^3^
*Z* = 2
Mo *K*α radiation
μ = 0.17 mm^−1^
*T* = 293 (2) K
0.25 × 0.20 × 0.18 mm

#### Data collection

Bruker SMART CCD area-detector diffractometer
Absorption correction: none
6826 measured reflections
4783 independent reflections
2940 reflections with *I* > 2σ(*I*)
*R*_int_ = 0.031

#### Refinement

*R*[*F* ^2^ > 2σ(*F* ^2^)] = 0.061
*wR*(*F* ^2^) = 0.187
*S* = 1.04
4783 reflections
309 parameters
57 restraints
H-atom parameters constrained
Δρ_max_ = 0.48 e Å^−3^
Δρ_min_ = −0.32 e Å^−3^

### 

Data collection: *SMART* (Bruker, 1997 ▶); cell refinement: *SAINT* (Bruker, 1997 ▶); data reduction: *SAINT*; program(s) used to solve structure: *SHELXS97* (Sheldrick, 2008 ▶); program(s) used to refine structure: *SHELXL97* (Sheldrick, 2008 ▶); molecular graphics: *SHELXTL* (Sheldrick, 2008 ▶); software used to prepare material for publication: *SHELXTL*.

## Supplementary Material

Crystal structure: contains datablocks global, I. DOI: 10.1107/S1600536808037665/at2649sup1.cif

Structure factors: contains datablocks I. DOI: 10.1107/S1600536808037665/at2649Isup2.hkl

Additional supplementary materials:  crystallographic information; 3D view; checkCIF report

## Figures and Tables

**Fig. 1.**
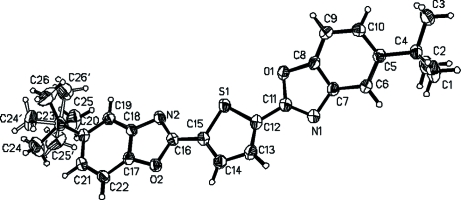
The structure of the title compound showing 30% probability displacement ellipsoids and the atom-numbering scheme.

## supplementary crystallographic information

### Comment

Fluorescent whitening agents have received considerable attention in the literature. They are attractive from several points of view in application (Chen *et al.*, 2008). As part of our search for new fluorescent whitening agent compounds we synthesized the title compound (I), and describe its structure here.

In (I) (Fig. 1), the C12—S1 bond length of 1.715 (3)Å is comparable with C—S bond [1.688 (2) Å] reported (Cowley *et al.*, 2002). The two 1,3-benzoxazole rings (N1/O1/C5-C11) and (N2/O2/C16-C22) are almost planar, with dihedral angles of 0.83 (18)° and 1.64 (17)°, respectively, between the five- and six-membered rings. The thiophene ring (S1/C12-C15) makes dihedral angles of 21.54 (19)° and 4.49 (18)°, respectively, with the five membered rings (O1/N1/C7/C8/C11) and (O2/N2/C16-C18).

In the crystal structure, there is no classical hydrogen bonds. The crystal packing is controlled by π-π stacking interactions involving the thiophene (Cg1: S1/C12-C15) and benzene (Cg2^i^: C17-C22) [ (i) 2 - x, 1 - y, - z] rings, with a centroid-centroid distance of 3.748 (2)Å.

### Experimental

A mixture of the thiophene-2,5-dicarboxylic acid (0.05 mol), and 4-*tert*-butyl-2-aminophenol (0.1 mol) was stirred in refluxing toluene (20 mL) for 4 h to afford the title compound (0.086 mol, yield 86%). Single crystals suitable for X-ray measurements were obtained by recrystallization from ethanol at room temperature.

### Refinement

H atoms were fixed geometrically and allowed to ride on their attached atoms, with C—H distances = 0.93 and- 0.96 Å, and with *U*_iso_=1.2 or 1.5*U*_eq_.

### Crystal data

**Table:**

C_26_H_26_N_2_O_2_S	*Z* = 2
*M**_r_* = 430.56	*F*_000_ = 456
Triclinic, *P*1	*D*_x_ = 1.280 Mg m^−^^3^
Hall symbol: -P 1	Mo *K*α radiation λ = 0.71073 Å
*a* = 6.0852 (12) Å	Cell parameters from 1520 reflections
*b* = 11.520 (2) Å	θ = 2.5–23.6º
*c* = 16.986 (3) Å	µ = 0.17 mm^−^^1^
α = 72.79 (3)º	*T* = 293 (2) K
β = 88.88 (3)º	Block, yellow
γ = 79.32 (3)º	0.25 × 0.20 × 0.18 mm
*V* = 1116.9 (4) Å^3^	

### Data collection

**Table:**

Bruker SMART CCD area-detector diffractometer	2940 reflections with *I* > 2σ(*I*)
Radiation source: fine-focus sealed tube	*R*_int_ = 0.031
Monochromator: graphite	θ_max_ = 27.0º
*T* = 293(2) K	θ_min_ = 1.9º
φ and ω scans	*h* = −7→7
Absorption correction: none	*k* = −14→14
6826 measured reflections	*l* = −21→17
4783 independent reflections	

### Refinement

**Table:**

Refinement on *F*^2^	Secondary atom site location: difference Fourier map
Least-squares matrix: full	Hydrogen site location: inferred from neighbouring sites
*R*[*F*^2^ > 2σ(*F*^2^)] = 0.061	H-atom parameters constrained
*wR*(*F*^2^) = 0.187	*w* = 1/[σ^2^(*F*_o_^2^) + (0.083*P*)^2^ + 0.378*P*] where *P* = (*F*_o_^2^ + 2*F*_c_^2^)/3
*S* = 1.04	(Δ/σ)_max_ < 0.001
4783 reflections	Δρ_max_ = 0.48 e Å^−^^3^
309 parameters	Δρ_min_ = −0.32 e Å^−^^3^
57 restraints	Extinction correction: SHELXL97 (Sheldrick, 2008), Fc^*^=kFc[1+0.001xFc^2^λ^3^/sin(2θ)]^-1/4^
Primary atom site location: structure-invariant direct methods	Extinction coefficient: 0.026 (4)

### Special details

**Table:**

Geometry. All e.s.d.'s (except the e.s.d. in the dihedral angle between two l.s. planes) are estimated using the full covariance matrix. The cell e.s.d.'s are taken into account individually in the estimation of e.s.d.'s in distances, angles and torsion angles; correlations between e.s.d.'s in cell parameters are only used when they are defined by crystal symmetry. An approximate (isotropic) treatment of cell e.s.d.'s is used for estimating e.s.d.'s involving l.s. planes.
Refinement. Refinement of *F*^2^ against ALL reflections. The weighted *R*-factor *wR* and goodness of fit *S* are based on *F*^2^, conventional *R*-factors *R* are based on *F*, with *F* set to zero for negative *F*^2^. The threshold expression of *F*^2^ > σ(*F*^2^) is used only for calculating *R*-factors(gt) *etc*. and is not relevant to the choice of reflections for refinement. *R*-factors based on *F*^2^ are statistically about twice as large as those based on *F*, and *R*- factors based on ALL data will be even larger.

### Fractional atomic coordinates and isotropic or equivalent isotropic displacement parameters (Å^2^)

**Table:**

	*x*	*y*	*z*	*U*_iso_*/*U*_eq_	Occ. (<1)
S1	0.58719 (14)	0.77701 (8)	0.00117 (5)	0.0601 (3)	
O1	0.2519 (4)	1.00117 (19)	−0.08466 (12)	0.0567 (7)	
O2	1.1731 (3)	0.55542 (18)	0.07064 (12)	0.0517 (7)	
N1	0.3847 (5)	1.0267 (3)	−0.21200 (16)	0.0621 (9)	
N2	0.8447 (4)	0.5523 (2)	0.13353 (16)	0.0568 (9)	
C1	−0.1548 (7)	1.4283 (4)	−0.4007 (3)	0.0955 (17)	
C2	−0.3823 (7)	1.2682 (4)	−0.3939 (2)	0.0812 (14)	
C3	−0.4984 (8)	1.4230 (4)	−0.3198 (3)	0.0971 (17)	
C4	−0.2957 (6)	1.3431 (3)	−0.34509 (19)	0.0553 (10)	
C5	−0.1536 (5)	1.2540 (3)	−0.27123 (18)	0.0509 (10)	
C6	0.0494 (5)	1.1872 (3)	−0.28414 (18)	0.0563 (10)	
C7	0.1771 (5)	1.1053 (3)	−0.21673 (18)	0.0513 (10)	
C8	0.0973 (5)	1.0894 (3)	−0.13912 (17)	0.0502 (10)	
C9	−0.1032 (6)	1.1522 (3)	−0.1232 (2)	0.0639 (11)	
C10	−0.2260 (6)	1.2345 (3)	−0.19051 (19)	0.0589 (11)	
C11	0.4174 (5)	0.9704 (3)	−0.13440 (18)	0.0522 (10)	
C12	0.6087 (5)	0.8788 (3)	−0.09425 (18)	0.0520 (10)	
C13	0.8153 (6)	0.8580 (3)	−0.12375 (19)	0.0567 (11)	
C14	0.9600 (6)	0.7598 (3)	−0.06945 (19)	0.0549 (11)	
C15	0.8597 (5)	0.7069 (3)	0.00045 (18)	0.0506 (10)	
C16	0.9503 (5)	0.6044 (3)	0.07071 (19)	0.0501 (10)	
C17	1.2069 (5)	0.4605 (3)	0.14351 (17)	0.0472 (9)	
C18	1.0079 (5)	0.4580 (3)	0.18216 (18)	0.0482 (10)	
C19	0.9930 (5)	0.3697 (3)	0.25628 (18)	0.0545 (10)	
C20	1.1818 (5)	0.2826 (3)	0.29081 (18)	0.0506 (10)	
C21	1.3811 (5)	0.2891 (3)	0.24862 (19)	0.0562 (11)	
C22	1.4005 (5)	0.3778 (3)	0.17496 (19)	0.0543 (10)	
C23	1.1718 (5)	0.1827 (3)	0.37112 (19)	0.0642 (11)	
C24	1.3987 (10)	0.1150 (10)	0.4102 (6)	0.163 (4)	0.539 (8)
C25	1.0524 (16)	0.0853 (6)	0.3523 (4)	0.098 (3)	0.539 (8)
C26	1.0314 (17)	0.2331 (7)	0.4322 (4)	0.112 (4)	0.539 (8)
C26'	0.9370 (10)	0.1644 (12)	0.3948 (8)	0.160 (5)	0.461 (8)
C24'	1.273 (2)	0.2224 (8)	0.4398 (5)	0.104 (4)	0.461 (8)
C25'	1.3145 (18)	0.0592 (6)	0.3717 (6)	0.111 (4)	0.461 (8)
H1A	−0.24570	1.48400	−0.44690	0.1440*	
H1B	−0.03250	1.37960	−0.42020	0.1440*	
H1C	−0.09720	1.47480	−0.37020	0.1440*	
H2A	−0.47220	1.32330	−0.44060	0.1220*	
H3B	−0.44820	1.47120	−0.28870	0.1450*	
H3C	−0.59200	1.37060	−0.28640	0.1450*	
H6A	0.10010	1.19690	−0.33730	0.0680*	
H9A	−0.15400	1.14010	−0.06990	0.0760*	
H2B	−0.47120	1.21410	−0.35930	0.1220*	
H2C	−0.25800	1.21990	−0.41260	0.1220*	
H3A	−0.58200	1.47730	−0.36820	0.1450*	
H19A	0.85790	0.36850	0.28280	0.0650*	
H21A	1.50760	0.23040	0.27150	0.0680*	
H22A	1.53530	0.38140	0.14850	0.0650*	
H24A	1.44840	0.04530	0.39010	0.2450*	0.539 (8)
H24B	1.38780	0.08690	0.46900	0.2450*	0.539 (8)
H24C	1.50390	0.17000	0.39630	0.2450*	0.539 (8)
H25A	0.99670	0.11480	0.29600	0.1460*	0.539 (8)
H25B	0.93010	0.07220	0.38830	0.1460*	0.539 (8)
H25C	1.15680	0.00880	0.36080	0.1460*	0.539 (8)
H26A	0.87580	0.23900	0.41970	0.1680*	0.539 (8)
H26B	1.05960	0.31370	0.42880	0.1680*	0.539 (8)
H26C	1.06940	0.17870	0.48700	0.1680*	0.539 (8)
H10A	−0.36330	1.27920	−0.18190	0.0710*	
H13A	0.85680	0.90420	−0.17470	0.0680*	
H14A	1.10850	0.73370	−0.08010	0.0660*	
H24D	1.33780	0.29410	0.41540	0.1560*	0.461 (8)
H24E	1.38710	0.15600	0.47090	0.1560*	0.461 (8)
H24F	1.15780	0.24140	0.47580	0.1560*	0.461 (8)
H25D	1.24530	0.02460	0.33590	0.1670*	0.461 (8)
H25E	1.32770	0.00360	0.42680	0.1670*	0.461 (8)
H25F	1.46060	0.07150	0.35280	0.1670*	0.461 (8)
H26D	0.86350	0.14870	0.35050	0.2400*	0.461 (8)
H26E	0.85490	0.23750	0.40530	0.2400*	0.461 (8)
H26F	0.94360	0.09520	0.44350	0.2400*	0.461 (8)

### Atomic displacement parameters (Å^2^)

**Table:**

	*U*^11^	*U*^22^	*U*^33^	*U*^12^	*U*^13^	*U*^23^
S1	0.0560 (5)	0.0611 (5)	0.0504 (5)	−0.0031 (4)	−0.0010 (4)	−0.0020 (4)
O2	0.0479 (12)	0.0515 (11)	0.0502 (12)	−0.0087 (9)	−0.0014 (9)	−0.0071 (9)
O1	0.0662 (14)	0.0563 (12)	0.0404 (11)	−0.0022 (11)	−0.0055 (10)	−0.0086 (9)
N1	0.0601 (17)	0.0672 (17)	0.0474 (15)	0.0009 (14)	−0.0021 (12)	−0.0072 (13)
N2	0.0476 (15)	0.0606 (15)	0.0517 (15)	−0.0058 (12)	−0.0029 (12)	−0.0030 (12)
C1	0.096 (3)	0.072 (3)	0.093 (3)	−0.018 (2)	−0.024 (2)	0.017 (2)
C2	0.091 (3)	0.079 (2)	0.073 (2)	−0.004 (2)	−0.028 (2)	−0.027 (2)
C3	0.101 (3)	0.095 (3)	0.081 (3)	0.036 (3)	−0.025 (2)	−0.036 (2)
C4	0.062 (2)	0.0510 (17)	0.0498 (17)	−0.0043 (15)	−0.0088 (15)	−0.0136 (14)
C5	0.060 (2)	0.0474 (16)	0.0457 (17)	−0.0102 (14)	−0.0036 (14)	−0.0142 (13)
C6	0.063 (2)	0.0590 (18)	0.0402 (16)	−0.0035 (16)	0.0005 (14)	−0.0094 (13)
C7	0.0578 (19)	0.0460 (16)	0.0454 (17)	−0.0063 (14)	0.0001 (14)	−0.0087 (13)
C8	0.063 (2)	0.0462 (16)	0.0393 (16)	−0.0076 (14)	−0.0022 (14)	−0.0107 (12)
C9	0.073 (2)	0.071 (2)	0.0438 (17)	−0.0045 (18)	0.0058 (16)	−0.0170 (15)
C10	0.061 (2)	0.0587 (19)	0.0548 (19)	−0.0007 (16)	−0.0010 (15)	−0.0200 (15)
C11	0.0556 (19)	0.0516 (17)	0.0470 (17)	−0.0095 (14)	−0.0017 (14)	−0.0110 (14)
C12	0.058 (2)	0.0505 (17)	0.0451 (16)	−0.0104 (14)	−0.0066 (14)	−0.0098 (13)
C13	0.062 (2)	0.0580 (18)	0.0468 (17)	−0.0165 (16)	−0.0009 (15)	−0.0071 (14)
C14	0.0524 (19)	0.0587 (18)	0.0517 (18)	−0.0119 (15)	0.0000 (14)	−0.0126 (14)
C15	0.0501 (18)	0.0497 (16)	0.0481 (17)	−0.0081 (14)	−0.0054 (14)	−0.0089 (13)
C16	0.0460 (17)	0.0499 (16)	0.0522 (18)	−0.0063 (14)	−0.0045 (14)	−0.0130 (14)
C17	0.0506 (18)	0.0442 (15)	0.0450 (16)	−0.0111 (13)	−0.0034 (13)	−0.0089 (12)
C18	0.0430 (17)	0.0492 (16)	0.0488 (17)	−0.0039 (13)	−0.0017 (13)	−0.0117 (13)
C19	0.0480 (18)	0.0606 (18)	0.0500 (17)	−0.0110 (15)	0.0031 (14)	−0.0086 (14)
C20	0.0497 (18)	0.0504 (16)	0.0494 (17)	−0.0081 (14)	−0.0056 (14)	−0.0119 (13)
C21	0.0496 (18)	0.0532 (18)	0.0602 (19)	−0.0018 (14)	−0.0120 (15)	−0.0121 (15)
C22	0.0417 (17)	0.0567 (18)	0.0612 (19)	−0.0060 (14)	0.0003 (14)	−0.0143 (15)
C23	0.063 (2)	0.062 (2)	0.0541 (19)	−0.0060 (17)	−0.0094 (16)	0.0005 (15)
C24	0.120 (7)	0.187 (9)	0.111 (7)	−0.025 (7)	−0.024 (6)	0.062 (6)
C25	0.149 (8)	0.065 (4)	0.072 (5)	−0.040 (5)	0.013 (5)	0.002 (3)
C26	0.182 (9)	0.084 (5)	0.059 (4)	−0.017 (6)	0.040 (5)	−0.013 (4)
C24'	0.168 (9)	0.087 (6)	0.051 (4)	−0.022 (6)	−0.015 (5)	−0.010 (4)
C25'	0.178 (10)	0.046 (4)	0.091 (6)	0.001 (5)	0.022 (6)	−0.007 (4)
C26'	0.105 (7)	0.175 (10)	0.143 (9)	−0.048 (7)	0.010 (7)	0.053 (7)

### Geometric parameters (Å, °)

**Table:**

S1—C15	1.706 (3)	C14—H14A	0.9300
S1—C12	1.715 (3)	C15—C16	1.440 (4)
O2—C16	1.369 (3)	C17—C18	1.367 (4)
O2—C17	1.377 (3)	C17—C22	1.374 (4)
O1—C11	1.366 (4)	C18—C19	1.379 (4)
O1—C8	1.377 (3)	C19—C20	1.386 (4)
N1—C11	1.286 (4)	C19—H19A	0.9300
N1—C7	1.400 (4)	C20—C21	1.399 (4)
N2—C16	1.284 (4)	C20—C23	1.511 (4)
N2—C18	1.397 (4)	C21—C22	1.380 (4)
C1—C4	1.524 (5)	C21—H21A	0.9300
C1—H1A	0.9600	C22—H22A	0.9300
C1—H1B	0.9600	C23—C26	1.511 (4)
C1—H1C	0.9600	C23—C26'	1.512 (5)
C2—C4	1.523 (5)	C23—C24	1.512 (5)
C2—H2A	0.9600	C23—C25'	1.521 (5)
C2—H2B	0.9600	C23—C24'	1.550 (5)
C2—H2C	0.9600	C23—C25	1.553 (4)
C3—C4	1.531 (5)	C24—H24A	0.9600
C3—H3A	0.9600	C24—H24B	0.9600
C3—H3B	0.9600	C24—H24C	0.9600
C3—H3C	0.9600	C25—H25A	0.9600
C4—C5	1.526 (4)	C25—H25B	0.9600
C5—C6	1.379 (4)	C25—H25C	0.9600
C5—C10	1.398 (4)	C26—H26A	0.9600
C6—C7	1.389 (4)	C26—H26B	0.9600
C6—H6A	0.9300	C26—H26C	0.9600
C7—C8	1.368 (4)	C24'—H24D	0.9600
C8—C9	1.365 (5)	C24'—H24E	0.9600
C9—C10	1.376 (4)	C24'—H24F	0.9600
C9—H9A	0.9300	C25'—H25D	0.9600
C10—H10A	0.9300	C25'—H25E	0.9600
C11—C12	1.444 (4)	C25'—H25F	0.9600
C12—C13	1.350 (4)	C26'—H26D	0.9600
C13—C14	1.397 (4)	C26'—H26E	0.9600
C13—H13A	0.9300	C26'—H26F	0.9600
C14—C15	1.355 (4)		
			
C15—S1—C12	90.76 (15)	C19—C18—N2	130.7 (3)
C16—O2—C17	103.0 (2)	C18—C19—C20	119.2 (3)
C11—O1—C8	103.4 (2)	C18—C19—H19A	120.4
C11—N1—C7	103.6 (3)	C20—C19—H19A	120.4
C16—N2—C18	104.0 (3)	C19—C20—C21	118.0 (3)
C4—C1—H1A	109.5	C19—C20—C23	120.9 (3)
C4—C1—H1B	109.5	C21—C20—C23	121.1 (3)
H1A—C1—H1B	109.5	C22—C21—C20	123.7 (3)
C4—C1—H1C	109.5	C22—C21—H21A	118.1
H1A—C1—H1C	109.5	C20—C21—H21A	118.1
H1B—C1—H1C	109.5	C17—C22—C21	115.4 (3)
C4—C2—H2A	109.5	C17—C22—H22A	122.3
C4—C2—H2B	109.5	C21—C22—H22A	122.3
H2A—C2—H2B	109.5	C26—C23—C20	111.4 (4)
C4—C2—H2C	109.5	C26—C23—C26'	53.4 (5)
H2A—C2—H2C	109.5	C20—C23—C26'	113.9 (5)
H2B—C2—H2C	109.5	C26—C23—C24	110.0 (3)
C4—C3—H3A	109.5	C20—C23—C24	114.0 (5)
C4—C3—H3B	109.5	C26'—C23—C24	132.0 (6)
H3A—C3—H3B	109.5	C26—C23—C25'	136.2 (5)
C4—C3—H3C	109.5	C20—C23—C25'	112.4 (4)
H3A—C3—H3C	109.5	C26'—C23—C25'	109.2 (4)
H3B—C3—H3C	109.5	C24—C23—C25'	47.4 (4)
C2—C4—C1	108.9 (3)	C26—C23—C24'	56.9 (4)
C2—C4—C5	108.7 (3)	C20—C23—C24'	107.2 (4)
C1—C4—C5	110.1 (3)	C26'—C23—C24'	107.4 (4)
C2—C4—C3	107.8 (3)	C24—C23—C24'	60.3 (4)
C1—C4—C3	108.6 (3)	C25'—C23—C24'	106.3 (4)
C5—C4—C3	112.7 (3)	C26—C23—C25	106.7 (3)
C6—C5—C10	118.6 (3)	C20—C23—C25	107.4 (3)
C6—C5—C4	119.4 (3)	C26'—C23—C25	54.6 (5)
C10—C5—C4	122.0 (3)	C24—C23—C25	107.0 (3)
C5—C6—C7	119.1 (3)	C25'—C23—C25	62.3 (4)
C5—C6—H6A	120.4	C24'—C23—C25	145.2 (5)
C7—C6—H6A	120.4	C23—C24—H24A	109.5
C8—C7—C6	119.8 (3)	C23—C24—H24B	109.5
C8—C7—N1	109.2 (3)	H24A—C24—H24B	109.5
C6—C7—N1	131.1 (3)	C23—C24—H24C	109.5
C9—C8—C7	123.4 (3)	H24A—C24—H24C	109.5
C9—C8—O1	128.8 (3)	H24B—C24—H24C	109.5
C7—C8—O1	107.9 (3)	C23—C25—H25A	109.5
C8—C9—C10	116.1 (3)	C23—C25—H25B	109.5
C8—C9—H9A	121.9	H25A—C25—H25B	109.5
C10—C9—H9A	121.9	C23—C25—H25C	109.5
C9—C10—C5	123.0 (3)	H25A—C25—H25C	109.5
C9—C10—H10A	118.5	H25B—C25—H25C	109.5
C5—C10—H10A	118.5	C23—C26—H26A	109.5
N1—C11—O1	116.0 (3)	C23—C26—H26B	109.5
N1—C11—C12	127.4 (3)	H26A—C26—H26B	109.5
O1—C11—C12	116.6 (3)	C23—C26—H26C	109.5
C13—C12—C11	127.6 (3)	H26A—C26—H26C	109.5
C13—C12—S1	111.9 (2)	H26B—C26—H26C	109.5
C11—C12—S1	120.4 (3)	C23—C24'—H24D	109.5
C12—C13—C14	112.7 (3)	C23—C24'—H24E	109.5
C12—C13—H13A	123.6	H24D—C24'—H24E	109.5
C14—C13—H13A	123.6	C23—C24'—H24F	109.5
C15—C14—C13	112.4 (3)	H24D—C24'—H24F	109.5
C15—C14—H14A	123.8	H24E—C24'—H24F	109.5
C13—C14—H14A	123.8	C23—C25'—H25D	109.5
C14—C15—C16	129.5 (3)	C23—C25'—H25E	109.5
C14—C15—S1	112.2 (2)	H25D—C25'—H25E	109.5
C16—C15—S1	118.3 (2)	C23—C25'—H25F	109.5
N2—C16—O2	115.9 (3)	H25D—C25'—H25F	109.5
N2—C16—C15	127.2 (3)	H25E—C25'—H25F	109.5
O2—C16—C15	116.9 (3)	C23—C26'—H26D	109.5
C18—C17—C22	123.2 (3)	C23—C26'—H26E	109.5
C18—C17—O2	108.3 (2)	H26D—C26'—H26E	109.5
C22—C17—O2	128.5 (3)	C23—C26'—H26F	109.5
C17—C18—C19	120.5 (3)	H26D—C26'—H26F	109.5
C17—C18—N2	108.8 (3)	H26E—C26'—H26F	109.5
			
C2—C4—C5—C6	68.7 (4)	C12—S1—C15—C16	179.9 (2)
C1—C4—C5—C6	−50.5 (4)	C18—N2—C16—O2	−0.1 (4)
C3—C4—C5—C6	−171.8 (3)	C18—N2—C16—C15	179.1 (3)
C2—C4—C5—C10	−109.1 (4)	C17—O2—C16—N2	0.1 (3)
C1—C4—C5—C10	131.7 (4)	C17—O2—C16—C15	−179.2 (2)
C3—C4—C5—C10	10.4 (5)	C14—C15—C16—N2	−175.3 (3)
C10—C5—C6—C7	−1.5 (5)	S1—C15—C16—N2	5.0 (4)
C4—C5—C6—C7	−179.4 (3)	C14—C15—C16—O2	3.9 (5)
C5—C6—C7—C8	1.9 (5)	S1—C15—C16—O2	−175.8 (2)
C5—C6—C7—N1	179.6 (3)	C16—O2—C17—C18	0.0 (3)
C11—N1—C7—C8	−0.3 (4)	C16—O2—C17—C22	178.8 (3)
C11—N1—C7—C6	−178.1 (3)	C22—C17—C18—C19	−0.2 (5)
C6—C7—C8—C9	−1.2 (5)	O2—C17—C18—C19	178.7 (3)
N1—C7—C8—C9	−179.4 (3)	C22—C17—C18—N2	−178.9 (3)
C6—C7—C8—O1	178.0 (3)	O2—C17—C18—N2	0.0 (3)
N1—C7—C8—O1	−0.1 (3)	C16—N2—C18—C17	0.0 (3)
C11—O1—C8—C9	179.6 (3)	C16—N2—C18—C19	−178.5 (3)
C11—O1—C8—C7	0.4 (3)	C17—C18—C19—C20	−0.6 (5)
C7—C8—C9—C10	0.2 (5)	N2—C18—C19—C20	177.8 (3)
O1—C8—C9—C10	−179.0 (3)	C18—C19—C20—C21	0.4 (4)
C8—C9—C10—C5	0.2 (5)	C18—C19—C20—C23	−178.8 (3)
C6—C5—C10—C9	0.4 (5)	C19—C20—C21—C22	0.5 (5)
C4—C5—C10—C9	178.3 (3)	C23—C20—C21—C22	179.8 (3)
C7—N1—C11—O1	0.6 (4)	C18—C17—C22—C21	1.0 (4)
C7—N1—C11—C12	−179.3 (3)	O2—C17—C22—C21	−177.6 (3)
C8—O1—C11—N1	−0.6 (4)	C20—C21—C22—C17	−1.2 (5)
C8—O1—C11—C12	179.3 (3)	C19—C20—C23—C26	−41.2 (5)
N1—C11—C12—C13	20.3 (6)	C21—C20—C23—C26	139.6 (5)
O1—C11—C12—C13	−159.6 (3)	C19—C20—C23—C26'	17.0 (7)
N1—C11—C12—S1	−157.8 (3)	C21—C20—C23—C26'	−162.3 (6)
O1—C11—C12—S1	22.3 (4)	C19—C20—C23—C24	−166.3 (5)
C15—S1—C12—C13	0.1 (3)	C21—C20—C23—C24	14.5 (6)
C15—S1—C12—C11	178.5 (3)	C19—C20—C23—C25'	141.8 (5)
C11—C12—C13—C14	−178.5 (3)	C21—C20—C23—C25'	−37.4 (6)
S1—C12—C13—C14	−0.3 (4)	C19—C20—C23—C24'	−101.7 (5)
C12—C13—C14—C15	0.4 (4)	C21—C20—C23—C24'	79.0 (6)
C13—C14—C15—C16	179.9 (3)	C19—C20—C23—C25	75.3 (5)
C13—C14—C15—S1	−0.4 (4)	C21—C20—C23—C25	−104.0 (5)
C12—S1—C15—C14	0.2 (3)		
